# Simultaneous laparoscopic management of Morgagni hernia and cholelithiasis: two case reports

**DOI:** 10.1186/s13104-015-1249-y

**Published:** 2015-07-01

**Authors:** Vikal Chandra Shakya

**Affiliations:** Department of Surgery, Civil Service Hospital of Nepal, Kathmandu, Nepal

**Keywords:** Laparoscopy, Cholelithiasis, Morgagni hernia

## Abstract

**Background:**

Morgagni hernia is a rare type of diaphragmatic hernia. Though in the past, it has been dealt with an open approach, nowadays laparoscopic management is a favored approach. However, there are few controversies in this scenario.

**Case presentation:**

We present here two females of Aryan ethnicity, one 55 and another 45 years old, who presented with pain at upper abdomen and retrosternal chest pain; on investigations were found to have cholelithiasis along with Morgagni hernia which were managed via the laparoscopic approach in the same sitting.

**Conclusion:**

Repair of Morgagni hernia also via the minimally invasive technique can be offered to the patients like that for cholelithiasis.

## Background

Congenital diaphragmatic hernias are rare congenital defects in which the abdominal contents herniate through a defect in the diaphragm into the chest cavity. There are four different types: (1) anterolateral hernia; (2) posterolateral or Bochdalek hernia; (3) pars sternalis; and (4) anteromedial or Morgagni hernia. The latter is the least common variety, accounting for only 1–3% of all [1]. The foramen of Morgagni is a space in the retro-xiphoid sternocostal hiatus through which herniation of omentum, colon, stomach, or other viscera may occur [2]. It is usually asymptomatic and discovered incidentally on chest X-ray done for other pathologies. The usual treatment has been an open approach via thoracotomy or by laparotomy [2, 3]. We report two cases of successful simultaneous laparoscopic repair of Morgagni hernia and cholecystectomy, and provide a review of the literature with regard to laparoscopic repair in adults.

## Case presentation

### Case no 1

**Figure 1:**
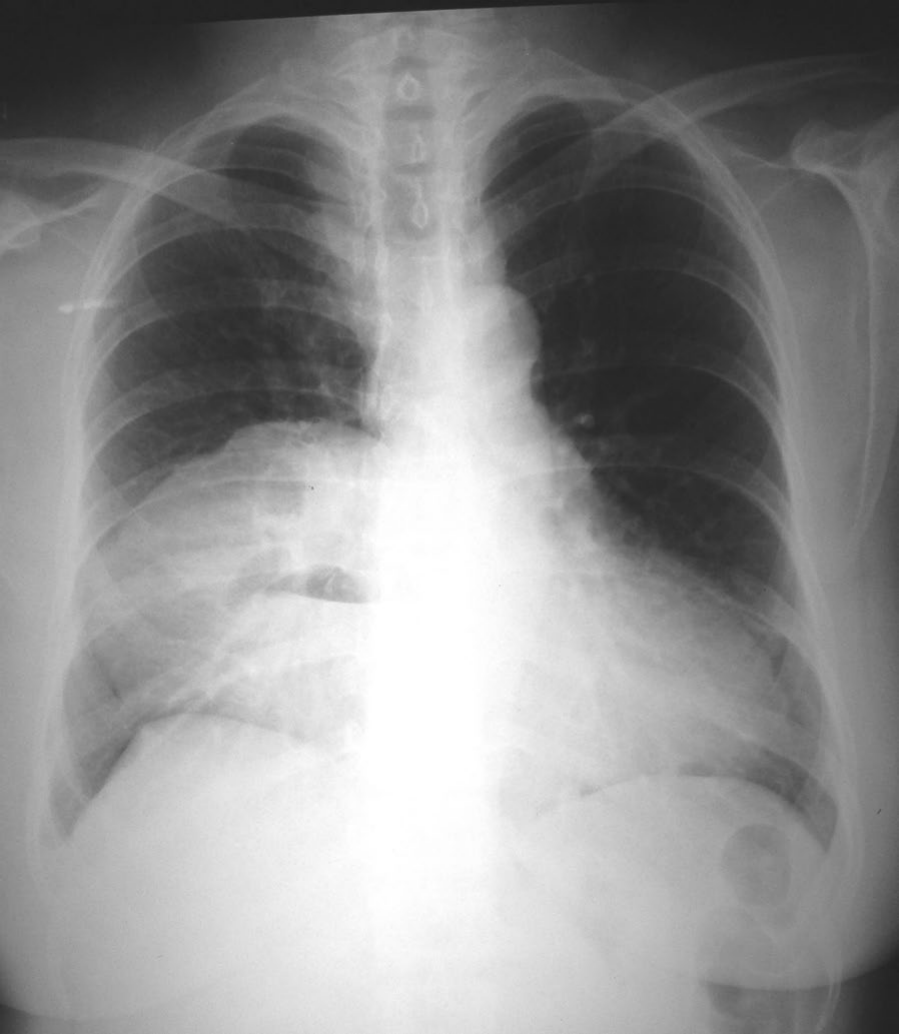
Preoperative chest radiograph of patient 1 showing right paracardiac shadow.

**Figure 2:**
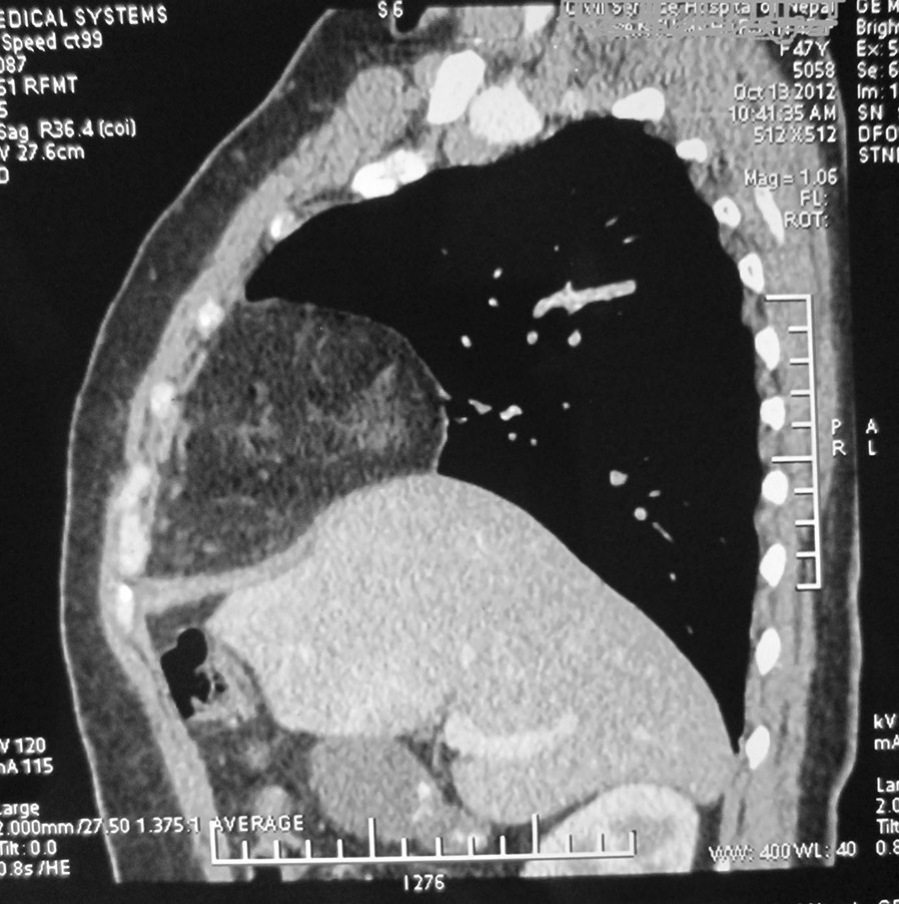
Preoperative computed tomography image of the thorax and abdomen in patient 1 showing Morgagni hernia anterior to the liver.

**Figure 3:**
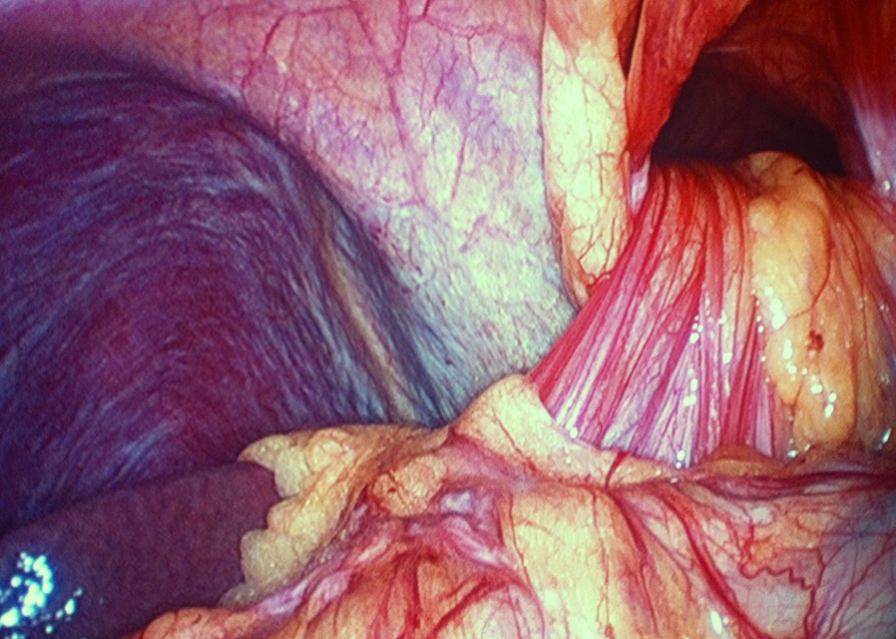
Intraoperative photograph showing the hernial defect with omental evisceration into the thorax.

A 55-year-old female of Aryan ethnicity had a history of vague epigastric pain more after taking meals for last 1 year. An ultrasound of the abdomen showed the presence of cholelithiasis. She was planned electively for a laparoscopic cholecystectomy at first. Preoperative workup was done, and no other abnormalities were found except in the chest X-ray, which revealed the presence of a right paracardiac shadow (Figure 1). Consequently, she was subjected to CT (computed tomography) scan of the thorax and upper abdomen, which showed the presence of omentum herniating through a retrosternal defect to lie in the right side of the heart in the thorax (Figure 2). After complete clinical and imaging evaluations, the patient was considered for a laparoscopic cholecystectomy and laparoscopic repair of the Morgagni hernia (Figure 3).

### Case no 2

**Figure 4:**
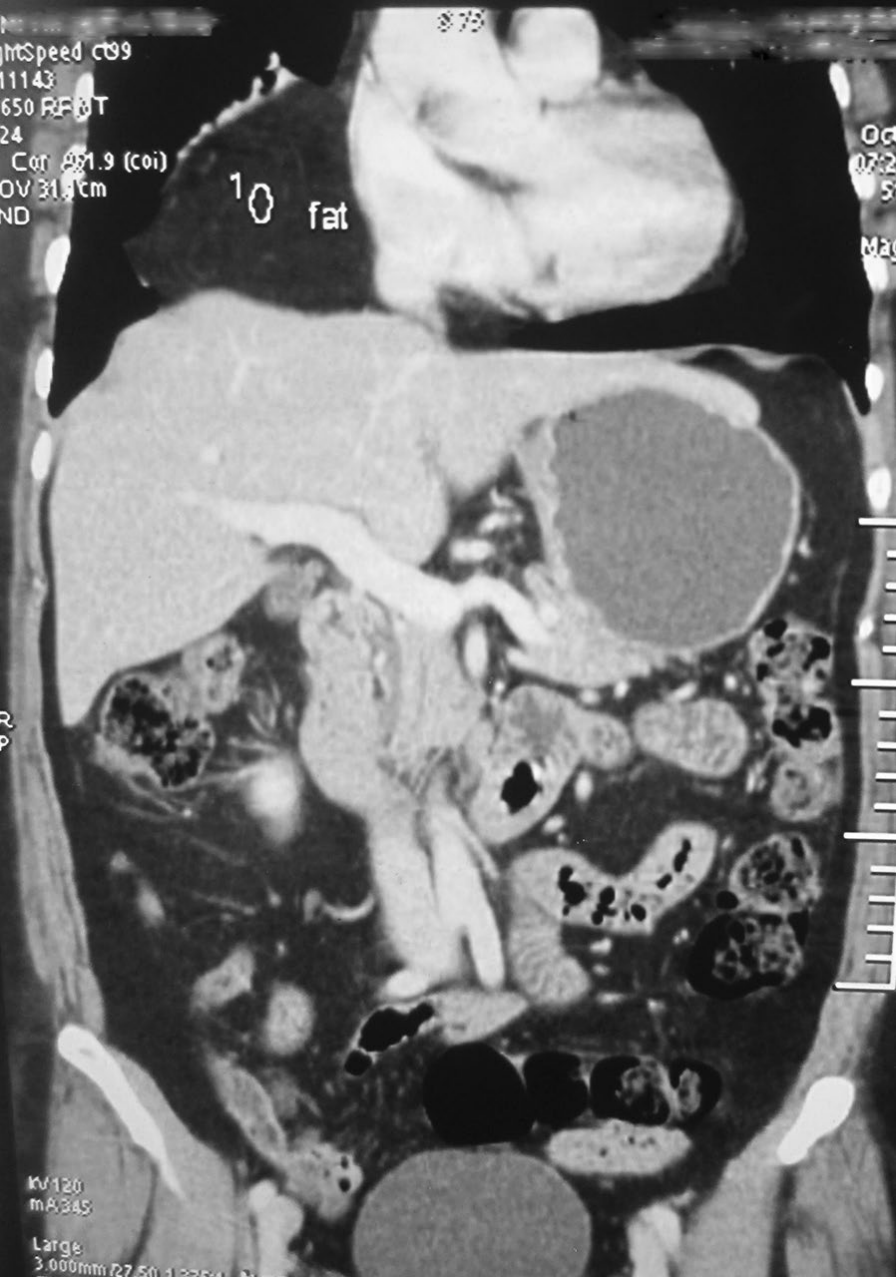
Preoperative computed tomography image of the thorax and abdomen in patient 2 showing Morgagni hernia.

Another 44-year-old female of Aryan ethnicity had complaints of retrosternal chest pain and heaviness, who was worked up as a cardiac case in other hospital. On further workup, she was found to have cholelithiasis on ultrasound of the abdomen, and was referred to our hospital for laparoscopic cholecystectomy. A chest X-ray was done, which showed a similar right paracardiac shadow; she was then subjected to a spiral thoraco-abdominal CT scan, which proved the diagnosis of Morgagni hernia (Figure 4).

**Figure 5:**
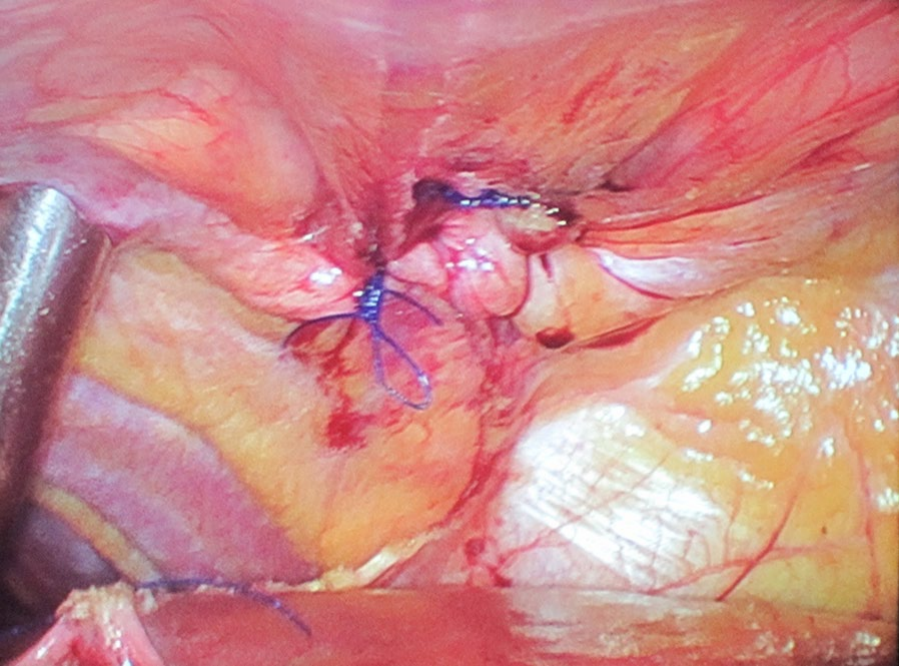
After reduction of the omentum, the hernial defect was obliterated with intracorporeal prolene sutures.

**Figure 6:**
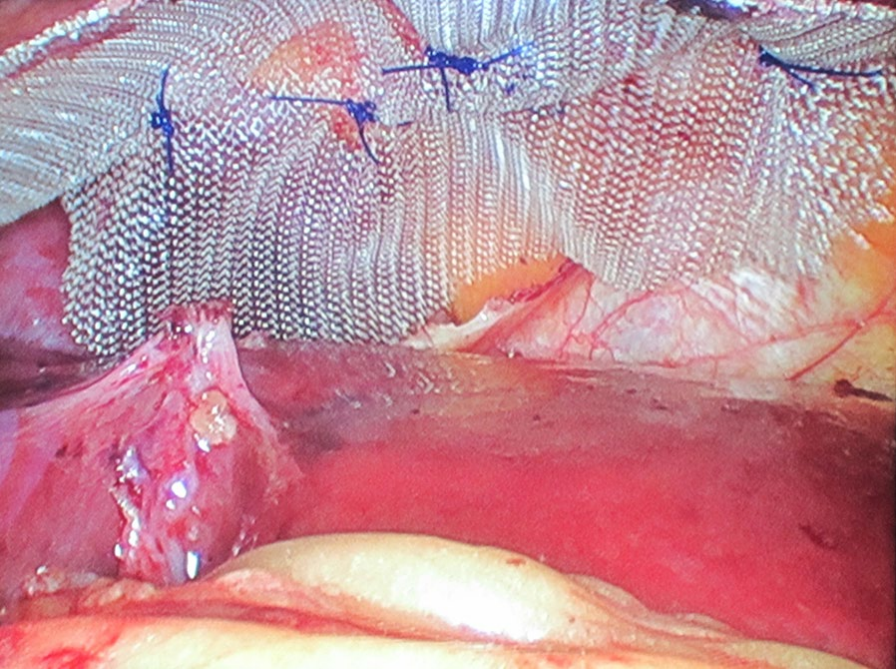
A polypropylene mesh was kept under the defect with wide overlapping and fixed with intracorporeal prolene sutures.

**Figure 7:**
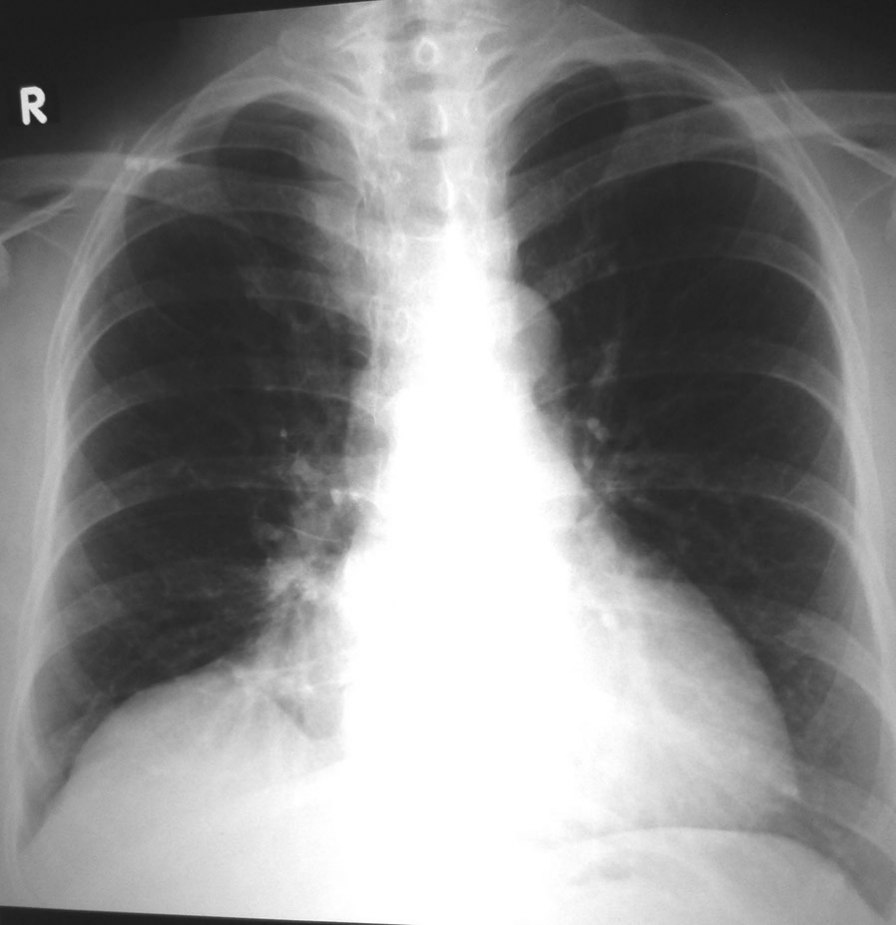
Postoperative chest radiograph showing good result.

For laparoscopic repair, general anesthesia was given, both patients were placed in the supine lithotomy position with their arms at their sides, Foley’s catheter was inserted. A 10-mm trocar port was inserted above the umbilicus via the open technique, and a 0^°^ camera was introduced into the abdominal cavity through this port, and CO_2_ pneumoperitoneum was created. Operating surgeon was positioned between the legs of the patients, and assistant surgeon at the left side. After primary evaluation in both patients, the Morgagni hernia was located: a segment of omentum from the transverse colon was seen herniating into the hernial sac (Figure 3). After insertion of two 5 mm trocar ports on the right and one 5 mm port on left upper abdominal quadrants respectively, patients’ position was changed to a reverse Trendelenburg; there were some adhesions of the omentum to the neck of the sac, and after division of these, the omentum was reduced back into the abdominal cavity. The falciform ligament was divided, and the edge of the defect was approximated with no. 1 polypropylene interrupted sutures (Figure 5). Next, the gallbladder was taken care of, after inserting a 10 mm epigastric port, and changing the position of the surgeon to left side of the patient. A polypropylene mesh of size 15 × 15 cm was inserted into the abdominal cavity through the epigastric 10 mm port; it was expanded over the defect and fixed to the anterior abdominal wall and edge of the diaphragmatic defect with a series of two rounds of intracorporeal prolene knots; care being taken not to take deep bites at the diaphragm and avoiding at the left cranial region (the region of the heart) (Figure 6). Altogether, for both procedures, total five ports were needed. Patients were admitted to the surgical intensive care unit for the first postoperative day and discharged 72 h after surgery; no postoperative complications were noted; both patients were discharged after proper ambulation and were on soft diets. Postoperative radiograph was satisfactory (Figure 7). Follow-up has been done for 12 months postoperatively; there has been no recurrence till date.

## Discussion

Hernia of Morgagni was first described by Giovanni Battista Morgagni, an Italian anatomist and pathologist in 1769, while performing a postmortem examination on a patient who died of a head injury [4]. The first laparoscopic repair was reported by Kuster et al. [5]. It is a rare form of diaphragmatic hernia that results from a congenital failure of the pars sternalis to fuse with the costal arches [6]. It is usually located on the right side, at the level of the seventh rib on either side of the xiphoid, in a space where the superior epigastric vessels pass; defects may also occur on the left, at the midline, or bilaterally, that on the left side is referred as a Larrey hernia [7].

Most patients with Morgagni hernia present later in life as a result of progressive changes in intra-abdominal pressure caused by pregnancy, obesity, and trauma and attenuation of the diaphragm from aging [3]. Patients may be asymptomatic or present with cardio-pulmonary symptoms due to compression of the thoracic organs or pulmonary infection. Differential diagnosis of this condition includes pleuropericardial cyst, pleural mesothelioma, pericardial fat pad, mediastinal lipoma, tumor or cyst of the diaphragm, thymoma, and anterior chest wall tumors [2]. The paracardiac shadow is usually found incidentally on chest radiographs, index of suspicion should be high, and diagnosis can be established with CT scan or magnetic resonance imaging. Surgical treatment is the choice because complications such as visceral strangulation have been described in the literature [8]. In our cases, Morgagni hernia was found along with cholelithiasis, and we decided to treat both conditions simultaneously via the minimally invasive approach. Probably this is the first report of its kind. It needed an additional port at the left upper quadrant, apart from the four ports needed for standard cholecystectomy, and a change in the position of the surgeons, viz. between the legs for repair of Morgagni hernia in lithotomy position and at the left side of the patient for cholelithiasis.

Since the first laparoscopic repair by Kuster et al. [5], more and more cases have been dealt with laparoscopy; laparoscopy is an excellent way to confirm diagnosis and to repair noncomplicated hernia of Morgagni. The hernial sac can be easily viewed through the laparoscope. Other advantages of laparoscopic repair are reduction in trauma, a faster recovery and faster return to normal diet and activity [9]. However, there are some controversies regarding important aspects in the laparoscopic repair [10]. One exists regarding the need for sac excision. There have been concerns against removal of the sac as this may result in massive pneumomediastinum and damage to the pericardium or mediastinal structures may occur during the dissection of the sac, which are life threatening [5]. Authors have suggested that excision of the sac may have the following advantages: (1) reduction of tissue trauma because only the sac is manipulated (rather than its contents) in cases where the colon or stomach are contained within the sac; (2) decreased chance for symptomatic fluid collection since the serous lining membrane is removed; and (3) sac excision negates the chance that the sac itself can act as a lead point for recurrent herniation [9]. However there is no available literature to recommend whether this influences the recurrence or formation of a cyst. In our case we did not attempt to remove the sac.

Another controversy exists as to whether prosthetic material should be used or not. In Morgagni hernia, the diaphragmatic musculature is weak and attenuated. Therefore, use of a suitable prosthesis would help in decreasing the recurrences, which may be possible with primary repair. Though in the literature, no patients with primary repair have had recurrences, still no long-term clinical or radiological follow-up has been recorded in these studies. Prostheses have been used in most cases, but again there is no uniformity; meshes used have included polypropylene mesh, and recent composite prostheses such as ePTFE (expanded polytetrafluoroethylene) mesh, porcine small intestine submucosa biocompatible prosthesis, parietex bilayered composite mesh and PVDF (polyvinylidene fluoride) mesh [11–14]. However, we decided to use polypropylene mesh, because of cost factor; and reperitonealization is generally not required as after the division of the falciform ligament, the liver comes to lie under the mesh and the chance of the mesh to adhere to hollow viscus is low. If needed, the mesh may be covered with omentum to avoid adhering of intra-abdominal viscera.

In surgical correction of Morgagni hernia, all the maneuvers involved in open surgery can be duplicated with laparoscopic technique, albeit with added advantages of minimal wound complications, good cosmetic results, less postoperative pain and a faster recovery. Though there are controversies regarding different aspects in laparoscopic repair, it is still difficult to advocate any one technique over the other based on the reported literatures, as no major complications or recurrences have been reported with these different methods.

## Conclusion

We believe with the advancements in laparoscopic repair, repair of Morgagni hernia also via the minimally invasive technique can be offered to the patients like that for cholelithiasis; that too in the same sitting if required.

### Consent

Written informed consent was obtained from both patients for publication of this Case Report and any accompanying images. A copy of the written consent is available for review by the Editor-in-Chief of this journal.

## Abbreviations

CT: computed tomography
CO2: carbon dioxide
ePTFE: expanded polytetrafluoroethylene
PVDF: polyvinylidene fluoride
